# Turning the page on front matter: Wishing Nan Eckardt a joyful retirement

**DOI:** 10.1093/plcell/koae241

**Published:** 2024-09-25

**Authors:** Ralph S Quatrano, Richard A Jorgensen, Cathie Martin, Sabeeha S Merchant, Blake C Meyers

**Affiliations:** Editor-in-Chief (1999–2003), *The Plant Cell*, American Society of Plant Biologists, USA; Emeritus Dean and Professor, Department of Biology, Washington University, St. Louis, MO 63130, USA; Editor-in-Chief (2004–2008), *The Plant Cell*, American Society of Plant Biologists, USA; Emeritus Professor, School of Plant Sciences, University of Arizona, Tucson, AZ 85721, USA; Editor-in-Chief (2009–2014), *The Plant Cell*, American Society of Plant Biologists, USA; Department of Metabolic Biology, John Innes Centre, Norwich NR4 7UH, UK; Editor-in-Chief (2014–2019), *The Plant Cell*, American Society of Plant Biologists, USA; Department of Plant and Microbial Biology and Department of Molecular and Cell Biology, University of California, Berkeley, CA 94720, USA; Editor-in-Chief (2020–2024), *The Plant Cell*, American Society of Plant Biologists, USA; The Genome Center, University of California, Davis, CA 95616, USA; Department of Plant Sciences, University of California, Davis, CA 95616, USA

It is with great appreciation and a touch of sadness that we mark the occasion of the retirement of Nancy (Nan) Eckardt, our long-serving Senior Features Editor at *The Plant Cell*. Nan has been an integral part of our editorial team for over two decades, bringing to the journal unparalleled dedication, expertise, and a passion for excellent science and writing.

Nan joined *The Plant Cell* in 2000 as the News and Reviews Editor (promoted to Senior Features Editor in 2009), and she has been a cornerstone of the editorial team. Nan received her PhD in Plant Physiology from Pennsylvania State University, with postdoctoral research at the University of Illinois, Oxford University, and Penn State, focusing on photosynthesis and plant responses to abiotic stress.

Over the years, Nan Eckardt has worked closely with the five Editors-in-Chief (EiCs) who have co-signed this letter; with those interactions, Nan brought unique contributions and innovations to *The Plant Cell*. Ralph Quatrano hired Nan in 2000, setting the stage for her career with *The Plant Cell* through her development and editing of “Front Matter”—the varied content that appears in the journal before the main research articles. Following Ralph, Rich Jorgensen worked with Nan to expand the editorial team by bringing in a team of Science Editors, which Nan led. She guided this team as they ultimately grew into an independent group of consultants, some of whom continued their excellent service to the journal. Cathie Martin was the third EiC to work with Nan, and this led to the development of the *Large-Scale Biology* section. Nan led this effort to turn a series of perspective articles on large-scale biology into a full-fledged category of research articles in 2011, and she has shepherded many of the submissions in this category through to publication. In Sabeeha Merchant's term as EiC, the Assistant Features Editors (AFEs) were introduced, adeptly managed, and edited by Nan (Eckardt et al. 2018). Nan's work with the AFEs continued with the current EiC, Blake Meyers, and Nan's responsibilities grew yet again to include a role in the assembly of Focus Issues and coordination of their many review articles. Each Editor-in-Chief has valued Nan's dedication and contributions, as she has left a legacy in the journal's pages and history.

In Nan's role as the Senior Features Editor, she has excelled in overseeing the scientific editing of accepted manuscripts, managing the peer review of review articles and letters to the editor, writing content for the front of the journal, and running *The Plant Cell* AFE program. Her commitment to clarity and precision has significantly improved the readability and accessibility of our articles, making complex plant biology topics understandable to a broader audience. By our count, Nan authored over 225 summaries of research papers (currently published as *In Brief* articles, originally as *In This Issue* summaries) in *The Plant Cell*, highlighting the work of our authors. Her work on these pieces started in 2000, with a highlight of two papers about the plant endomembrane system and the mechanisms of transport within the secretory pathway (Eckardt 2000).

Nan's contributions have extended beyond her editorial responsibilities. She has been a mentor and guide to many young scientists and editors, always willing to share her knowledge and experience. A ready smile and calm demeanor encouraged everyone to engage with her. Nan's passion for language and writing, combined with her deep love for plant biology, made her an invaluable asset to *The Plant Cell* and the broader plant science community. She's been a presence at many conferences, including ASPB's Plant Biology meetings, where she engages with attendees and presenters.

One of Nan's more recent initiatives was an invitation by *The Plant Cell* for members of the plant science community to contribute Letters to the Editor that address racism, bias, or discrimination in academia and STEM (Eckardt and Meyers 2020). This is an opportunity for authors to share their reflections, propose actionable steps, and contribute to the ongoing dialogue aimed at eradicating racism and promoting equity within the scientific community.

As Nan embarks on her well-deserved retirement, we reflect on the profound impact she has had on our journal and the field of plant biology. Her legacy of excellence in writing and dedication to the journal and society will continue to inspire us all.

Thank you, Nan, for your many years of outstanding service. We wish you all the best in your retirement and look forward to hearing about your new adventures.
